# TNF-α and LPA promote synergistic expression of COX-2 in human colonic myofibroblasts: role of LPA-mediated transactivation of upregulated EGFR

**DOI:** 10.1186/1471-230X-13-90

**Published:** 2013-05-20

**Authors:** James Yoo, Citlali Ekaterina Rodriguez Perez, Wenxian Nie, James Sinnett-Smith, Enrique Rozengurt

**Affiliations:** 1 Departments of Surgery and Medicine, David Geffen School of Medicine, CURE: Digestive Diseases Research Center, University of California, Los Angeles, CA, 90095, USA

**Keywords:** TNF-α, EGFR, COX-2, Myofibroblast

## Abstract

**Background:**

Enhanced EGF receptor (EGFR) signaling is a hallmark of many human cancers, though the role of enhanced EGFR signaling within the surrounding tumor stroma has not been well studied. The myofibroblast is an important stromal cell that demonstrates enhanced EGFR expression in the setting of inflammation, though the functional relevance is not known. We recently reported that TNF-α and the G protein-coupled receptor (GPCR) agonist lysophosphatidic acid (LPA) lead to synergistic cyclo-oxygenase-2 (COX-2) expression, an enzyme strongly associated with the development of colitis-associated cancer. Here, we investigate whether EGFR signaling plays a role in the synergistic COX-2 expression induced by LPA and TNF-α.

**Methods:**

18Co cells, a model of human colonic myofibroblasts, were grown to confluence on 35 × 10mm cell culture dishes and were used from passages 10–14. 18Co cells were treated with TNF-α (8.3 ng/ml) and LPA (10 μM). EGFR and COX-2 protein expression, Y1068 phosphorylation, and p42/44 MAPK phosphorylation were assessed by Western Blot, in the presence and absence of various inhibitors.

**Results:**

Exposure of 18Co cells to either TNF-α or LPA alone had no effect on EGFR autophosphorylation at Y1068. However, chronic exposure to TNF-α led to upregulation of EGFR in association with sustained LPA-mediated EGFR phosphorylation at Y1068. TNF-α and LPA also led to sustained p42/44 MAPK phosphorylation and synergistic COX-2 expression, effects that were partially inhibited by the EGFR tyrosine kinase inhibitor AG1478. p42/44 MAPK phosphorylation and COX-2 expression were inhibited to the same degree by the MMP inhibitors GM6001 and BB-94, suggesting that LPA-mediated EGFR transactivation involved MMP-mediated release of EGFR ligands from the cell surface. The Src inhibitor SU6556 inhibited TNF-α/LPA-mediated EGFR phosphorylation at Y1068, p42/44 MAPK phosphorylation, and COX-2 expression in a dose-dependent fashion, suggesting an upstream role of Src in the transactivation of EGFR.

**Conclusion:**

Synergistic COX-2 expression induced by TNF-α and LPA involves Src/MMP-mediated transactivation of EGFR and downstream p42/44 MAPK activation in human colonic myofibroblasts. Enhanced EGFR expression induced by TNF-α promotes GPCR-mediated EGFR transactivation in colonic myofibroblasts, providing an important mechanism for stromal COX-2 over-expression that may predispose to the development of colitis-associated cancer.

## Background

Myofibroblasts are an influential stromal subpopulation that interact with neighboring cells in a paracrine fashion to regulate a number of important cellular processes, including intestinal epithelial proliferation and differentiation along the crypt-villous axis, mucosal repair, and fibrosis [1]. They also participate in immune and inflammatory responses and have been implicated in the pathophysiology of inflammatory bowel disease (IBD) and colitis-associated cancer [2,3].

Myofibroblasts are a target of inflammatory mediator signaling and, in particular, TNF-α, a 17-kDa pro-inflammatory cytokine that has been implicated in the pathogenesis of colitis-associated cancer [4,5]. TNF-α is found in high concentrations in the lamina propria of patients with ulcerative colitis [6], which is where myofibroblasts reside, and is known to regulate the signaling pathways that govern their function [7,8]. TNF-α binds to its receptors, TNF-α receptor 1 (TNFR1) and TNF-α receptor 2 (TNFR2), triggering the formation of a multiprotein complex (TRADD, RIP, TRAF-2) that culminates in the activation of MAP kinases and the transcription factor NFκB [reviewed in [9]]. However, alternative cross talk mechanisms exist between TNF-α and other pro-inflammatory mediators, including multiple G protein-coupled receptor (GPCR)-mediated agonists [7,8], that not only sustain the chronic inflammation associated with IBD, but also trigger a counter-regulatory response intended to promote mucosal healing. Chronic inflammation leads to the upregulation of cyclo-oxygenase-2 (COX-2), also known as prostaglandin-endoperoxide synthase 2, which is encoded by the PTGS2 gene in humans. COX-2, the rate-limiting enzyme in the biosynthesis of prostaglandins (PGs) and thromboxanes, is one example of a cytoprotective response to mucosal injury that, when dysregulated, may promote epithelial transformation to an invasive phenotype and the development of cancer [8,10]. It is increasingly recognized that the stromal compartment is the major reservoir of COX-2 in the GI tract [11,12]. Therefore, the regulation of COX-2 expression in the colonic myofibroblast may be highly relevant to the pathogenesis of inflammation-associated cancer in the gut.

We recently reported that TNF-α dramatically upregulates epidermal growth factor receptor (EGFR) expression on the cell surface of colonic myofibroblasts [11], though the functional significance of this upregulated expression is not fully known. Here, we demonstrate, for the first time, that enhanced EGFR expression induced by TNF-α facilitates GPCR-mediated EGFR transactivation in colonic myofibroblasts, providing an important mechanism for stromal COX-2 over-expression that may predispose to the development of colitis-associated cancer.

## Methods

### Cell culture

18Co cells (CRL-1459) were purchased from American Type Culture Collection (Rockville, MD). These cells share structural and functional characteristics of *in situ* colonic myofibroblasts, including a reversible stellate morphology, α-smooth muscle actin (α-SMA) expression and the presence of multiple cell surface receptors [13]. 18Co cells were maintained at 37°C in Dulbecco’s modified Eagle’s medium (DMEM) supplemented with 10% fetal bovine serum in a humidified atmosphere containing 10% CO_2_ and 90% air. Cells were plated in 35 mm dishes (1 × 10^5^ cells/dish) and grown in DMEM containing 10% fetal bovine serum for 5–7 days until confluent, and used from passages 8–14.

### Western blot

Confluent 18Co cells, treated as indicated in the individual experiments, were lysed in 2× SDS-polyacrylamide gel electrophoresis (PAGE) sample buffer (20 mM Tris/HCl, pH 6.8, 6% SDS, 2 mM EDTA, 4% 2-mercaptoethanol, 10% glycerol) and boiled for 10 min. After SDS-PAGE, proteins were transferred to Immobilon-P membranes. The transfer was carried out at 100 V, 0.4A at 4°C for 5 h using a Bio-Rad transfer apparatus. The transfer buffer consisted of 200 mM glycine, 25 mM Tris, 0.01% SDS, and 20% CH_3_OH. Membranes were blocked and then incubated for 2 h with the desired antibodies diluted in PBS (pH 7.2) containing 3% nonfat dried milk. Primary antibodies bound to immunoreactive bands were visualized by enhanced chemiluminescence (ECL) detection with horseradish peroxidase-conjugated anti-mouse or anti-rabbit antibodies (GE Healthcare, Piscataway, NJ).

### Myofibroblast isolation

A protocol to obtain human tissue from surgical patients was approved by the UCLA Office of Human Research Protection Program (IRB #11-000337). Participation in this study involved obtaining written informed consent. Human colon tissue immediately taken from surgically resected colon was washed with ice cold sterile PBS and then shaken 5× for 15 min in HBSS containing 5 mM EDTA. Next, the tissue was incubated in 20 ml of RPMI-5 [RPMI with 5% FCS, 10 mM HEPES, 2 mM L-glutamine, 1 mM sodium pyruvate, 100 U/ml Pen-Strep] containing 10.5 mg of Dispase (GIBCO-Invitrogen, Carlsbad, CA) and 7.2 mg of collagenase D (Roche Diagnostics, Indianapolis, IN) for 2 h in a shaking 37°C incubator. The digested tissue was treated with ACK lysis buffer for 5 min, and then was passed through a 70-μM cell strainer into 100-mm dishes in RPMI-5. After a 3 h incubation, nonadherent cells were washed away, leaving adherent cells consisting mainly of macrophages and myofibroblasts. After several days, macrophages died off leaving cells with a myofibroblast phenotype that were consistently α-SMA and vimentin positive. Primary colonic myofibroblast cultures were used for experiments up to passage 4.

## Materials

TNF-α was purchased from R&D Systems (Minneapolis, MN). α-SMA antibody (1:1000, ab5694) was purchased from Abcam (Cambridge, MA). EGFR antibody (1:1000, #2232), Y1068 antibody (1:1000, #2234) and p42/44 MAPK antibody (1:1000, #9106) were purchased from Cell Signaling Technology (Danvers, MA). COX-2 antibody (1:1000, #160106) and LPA were purchased from Caymann Chemical (Ann Arbor, MI). GM6001, SU6556, and AG1478 were purchased from Calbiochem (Gibbstown, NJ). BB-94 was purchased from Tocris (Bristol, United Kingdom). EGF was purchased from Sigma-Aldrich (St. Louis, MO).

## Results and discussion

### TNF-α potentiates LPA-mediated EGFR phosphorylation at Y1068

**Figure 1:**
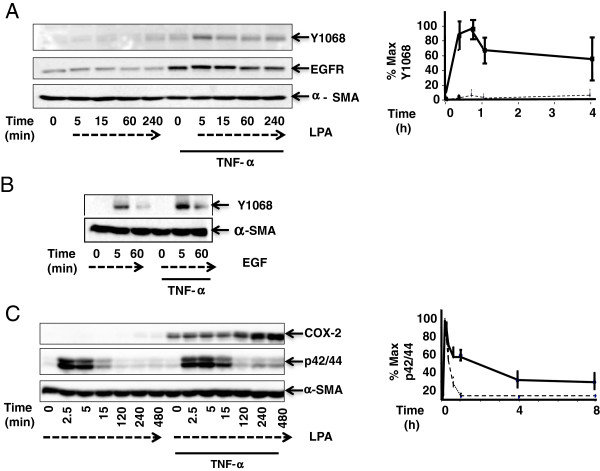
**TNF-α Potentiates LPA-mediated EGFR Phosphorylation at Y1068.** Panel **A**: Confluent 18Co cells were exposed to 10 μM LPA for various times (broken lines), with or without pre-incubation with 10 ng/ml TNF-α for 18 h (indicated by solid line). Western blot analysis was used with antibodies that detect EGFR and EGFR phosphorylation at Y1068. Densitometry analysis shows the mean ± S.E. (*n* ≥ 3), expressed as percentage of the maximal level of EGFR Y1068 phosphorylation. In all experiments, equal protein loading was verified using α-SMA antibody. Panel **B**: 18Co cells were incubated with EGF (5 ng/ml) for 5 or 60 min (broken lines), with or without pre-incubation with 10 ng/ml TNF-α for 18 h (indicated by solid line). Western blot analysis used an antibody detecting EGFR phosphorylation at Y1068. The results are representative of three separate experiments. Panel **C**: 18Co cells were exposed to 10 μM LPA for 8 h (broken lines), without or with pre-incubation with 10 ng/ml TNF-α for 18 h (indicated by solid line). Western blot analysis detected COX-2 and p42/44 MAPK phosphorylation. The results shown are the mean ± S.E. (*n* ≥ 3), expressed as a percentage of the maximal level of p42/44 MAPK phosphorylation, displayed graphically on the right.

To determine whether chronic exposure to TNF-α affects LPA-mediated transactivation of EGFR, 18Co cells were stimulated with LPA over 4 h, with or without a prior treatment with TNF-α for 18 h. EGFR auto-phosphorylation was monitored by Western blot analysis using an antibody that detects the phosphorylated state of the residue Y1068. In cells exposed to LPA alone, there was no detectable phosphorylation of EGFR at Y1068 (Figure 1A). In contrast, 18Co cells treated with TNF-α for 18 h displayed a striking increase in EGFR phosphorylation at Y1068, which correlated with an upward shift of the EGFR band. LPA-induced EGFR phosphorylation was rapid (evident after 5 min of stimulation) and sustained since it remained elevated over the 4 h time period. 18Co cells treated with TNF-α for 18 h displayed a marked increase in EGFR protein expression, consistent with previous results [11].

The response to LPA was notably different when compared to 18Co cells that were exposed to EGF (5 ng/ml), a direct EGFR ligand, with or without a prior incubation with TNF-α for 18 h (Figure 1B). Stimulation of 18Co cells with EGF alone led to a rapid and transient phosphorylation of EGFR at Y1068, returning close to baseline levels after 1 h. Pre-incubation with TNF-α for 18 h followed by exposure to EGF enhanced the signal intensity of Y1068 phosphorylation, consistent with increased EGFR protein expression, but did not significantly alter the time course, with a sharp decline in Y1068 phosphorylation levels at 1 h. These results demonstrate that while chronic exposure to TNF-α potentiates both EGF- and LPA-mediated EGFR phosphorylation at Y1068, the pattern of phosphorylation is strikingly different, with LPA producing a less pronounced but more sustained EGFR phosphorylation compared to that induced by direct ligand binding by EGF.

### Enhanced p42/44 MAPK phosphorylation and COX-2 expression induced by TNF-α and LPA are partially inhibited by AG1478

The sustained level of phosphorylation of EGFR induced by treatment with TNF-α and LPA suggested a potential role in long-term processes such as the regulation of COX-2 [8]. Stimulation of 18Co cells with LPA after treatment with TNF-α produced a synergistic time-dependent increase in COX-2 protein expression that was evident after 2 h and steadily increases over 8 h (Figure 1C), consistent with previous reports [8]. To determine whether p42/44 MAPK, a known downstream signaling target of both LPA and the EGFR, is involved in mediating this response, p42/44 MAPK phosphorylation was also analyzed by Western blot. Exposure of 18Co cells to LPA alone led to rapid and transient p42/44 MAPK phosphorylation, evident after 2.5 min with a return to baseline levels after 15 min, as shown in Figure 1C. However, in cells exposed to both TNF-α and LPA, there was a striking increase in the duration of p42/44 MAPK phosphorylation that was evident at 15 min and was sustained over 8 h. These results demonstrate that TNF-α and LPA stimulate synergistic COX-2 expression that is associated with enhanced p42/44 MAPK phosphorylation in human colonic myofibroblasts.

**Figure 2:**
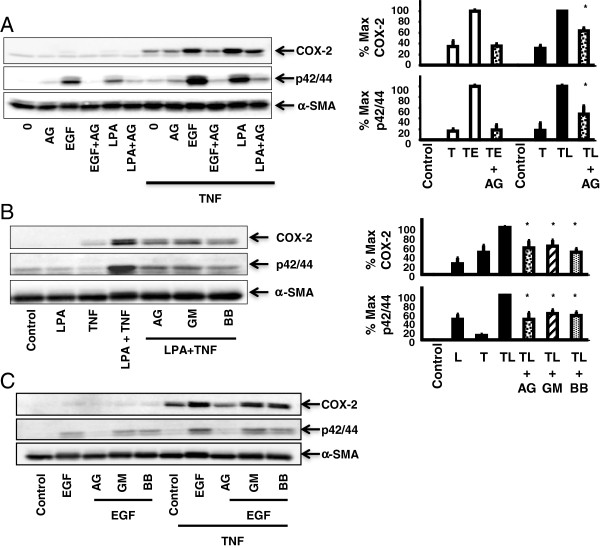
**Enhanced p42/44 MAPK phosphorylation and COX-2 expression induced by TNF-α and LPA are partially inhibited by AG1478 and MMP inhibitors.** Panel **A**: 18Co cells were pre-treated with 1 μM AG1478 for 1 h and then 5 ng/ml EGF or 10 μM LPA with or without pre-incubation with 10 ng/ml TNF-α for 18 h. Western blot analysis detected COX-2 and p42/44 MAPK phosphorylation. The results shown are the mean ± S.E. (*n* ≥ 3), expressed as a percentage of the maximal level of COX-2 expression and p42/44 MAPK phosphorylation, displayed graphically on the right. For all experiments, * denotes statistical significance, p < 0.05. Panel **B**: 18Co cells were incubated with or without 10 ng/ml TNF-α for 18 h, followed by 10 μM LPA for 4 h with or without pre-treatment with 1 μM AG1478, 10 μM GM6001, or 5 μM BB-94 for 1 h. The results shown are the mean ± S.E. (*n* ≥ 3), expressed as a percentage of the maximal level of COX-2 expression and p42/44 MAPK phosphorylation, displayed graphically on the right. Panel **C**: 18Co cells were incubated with or without 10 ng/ml TNF-α for 18 h, followed by exposure to 5 ng/ml EGF for 4 h with or without pre-treatment with 1 μM AG1478, 10 μM GM6001, or 5 μM BB-94 for 1 h. The results are representative of three separate experiments.

In order to evaluate a possible role of EGFR in the synergistic expression of COX-2 in response to LPA and TNF-α, 18Co cells were pre-incubated with the EGFR tyrosine kinase inhibitor AG1478 for 1 h prior to exposure to LPA with or without TNF-α. Cells exposed to TNF-α and LPA demonstrated enhanced COX-2 expression and p42/44 MAPK phosphorylation, as previously described (Figure 2A). AG1478 partially inhibited both p42/44 MAPK phosphorylation and COX-2 expression, suggesting that the EGFR and the downstream signaling target p42/44 MAPK were both involved in this response.

To confirm specificity for EGFR, 18Co cells were also pre-incubated with AG1478 and then exposed to EGF, which only binds to this receptor, with or without TNF-α. As anticipated, AG1478 inhibited EGF-mediated COX-2 expression and p42/44 MAPK phosphorylation to baseline levels (Figure 2A). Importantly, AG1478 did not inhibit COX-2 expression induced by TNF-α alone, suggesting that EGFR transactivation was not involved in TNF-α-mediated COX-2 expression. The data indicates that the maximal expression of COX-2 protein in response to TNF-α and LPA requires EGFR kinase activity.

### p42/44 MAPK phosphorylation and COX-2 expression induced by TNF-α and LPA are partially inhibited by MMP inhibitors

A potential mechanism for GPCR-induced EGFR transactivation involves matrix metalloproteinase (MMP)-activated release of extracellular ligands by the a disintegrin and metalloprotease (ADAM) family [14]. To test for the involvement of MMP’s in the expression of COX-2 induced by TNF-α and LPA, 18Co cells were treated with TNF-α and LPA in the presence or absence of the broad-spectrum MMP inhibitors GM6001 and BB-94, which inhibit multiple MMP’s and ADAMs. Both BB-94 and GM6001 partially inhibited COX-2 expression, and to the same degree as AG1478, suggesting that transactivation of EGFR was MMP-mediated (Figure 2B). The consistent findings seen with two different MMP inhibitors implies that the inhibitory effects were not due to off target effects. Accordingly, GM6001 and BB-94 did not inhibit COX-2 expression and p42/44 MAPK phosphorylation induced by TNF-α and EGF (Figure 2C).

### The Src inhibitor SU6556 partially inhibits EGFR phosphorylation at Y1068, p42/44 MAPK activation, and COX-2 Expression

**Figure 3:**
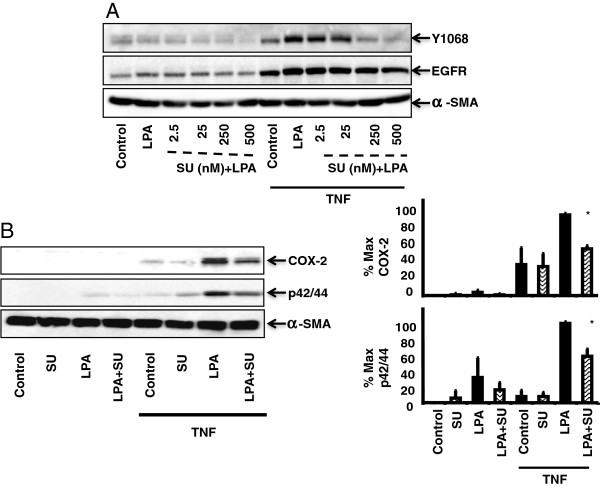
**The Src inhibitor SU6556 partially inhibits EGFR phosphorylation at Y1068, p42/44 MAPK activation, and COX-2 Expression.** Panel **A**: 18Co cells were incubated with or without 10 ng/ml TNF-α for 18 h, then were pre-treated with SU6556 for 1 h at the indicated concentrations, followed by 10 μM LPA for 4 h. The results are representative of three separate experiments. Panel **B**: 18Co cells were incubated for 18 h with or without 10 ng/ml TNF-α, then 250 nM SU6556 for 1 h followed by LPA for 4 h. Densitometry analysis is presented as mean ± S.E., (n ≥ 3), expressed as a percentage of the maximal level of COX-2 and p42/44 MAPK phosphorylation, depicted graphically on the right.

In some systems, MMP-induced EGFR transactivation involves Src, a non-receptor tyrosine kinase that has been implicated in EGFR phosphorylation [15]. To test for a role of Src in the transactivation of EGFR, 18Co cells were treated with TNF-α and LPA in the presence or absence of the Src inhibitor Su6556 at various concentrations. As demonstrated in Figure 3A, pre-treatment with SU6656 prevented Y1068 phosphorylation induced by TNF-α and LPA in a dose-dependent fashion. Having established the optimal concentration of SU6556, 18Co cells were pre-treated with 250 nM SU6556 followed by treatment with TNF-α and LPA. SU6656 inhibited p42/44 MAPK phosphorylation and COX-2 expression at levels that correlated to the degree of inhibition induced by AG1478, GM6001, and BB-94 (Figure 3B). To verify that SU6556 was not directly inhibiting EGFR auto-phosphorylation, 18Co cells were also treated with EGF and Y1068 phosphorylation was analyzed by Western blot. SU6556 did not inhibit EGF-induced EGFR phosphorylation (data not shown), suggesting that the inhibitory effects of SU6556 were not due to non-specific inhibition of EGFR. These results indicate that the full expression of COX-2 protein induced by TNF-α and LPA is partially mediated through Src-dependent transactivation of EGFR via MMP’s that result in downstream activation of p42/44 MAPK.

### Synergistic COX-2 expression induced by TNF-α and LPA involves EGFR Transactivation in Primary Human Colonic Myofibroblasts

**Figure 4:**
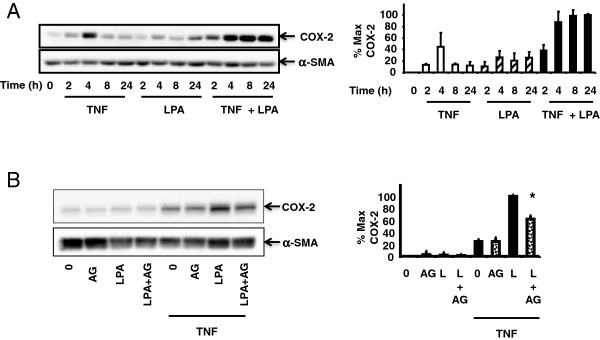
**Synergistic COX-2 expression induced by TNF-α and LPA involves EGFR Transactivation in Primary Human Colonic Myofibroblasts.** Panel **A**: Cell lysates of primary colonic myofibroblasts isolated from human colon tissue were incubated with 10 ng/ml TNF-α, 10 μM LPA, or both for 24 h. Western blot analysis used an antibody that detects COX-2. Densitometry analysis is presented as mean ± S.E. (n ≥ 3). Panel **B**: Primary colonic myofibroblasts were incubated with or without 10 ng/ml TNF-α for 18 h, then with AG1478 for 1 h followed by 10 μM LPA for 4 h. Western blot analysis detected COX-2. Densitometry analysis is presented as mean ± S.E., n ≥ 3.

To confirm that EGFR transactivation contributes to the enhanced expression of COX-2 induced by LPA and TNF-α is relevant and reproducible in early passage cultures of human cells, colonic myofibroblasts were isolated from surgically resected human colon tissue using a well-established protocol for myofibroblast isolation [16]. Isolated cells demonstrated a myofibroblast-like phenotype that was consistently α-SMA and vimentin positive [11]. As shown in Figure 4A, human colonic myofibroblasts exposed to TNF-α and LPA displayed a time-dependent increase in COX-2 expression over 24 h. Pre-incubation with AG1478 prior to exposure to LPA and TNF-α partially inhibited COX-2 expression (Figure 4B), supporting the contribution of EGFR transactivation in the enhanced expression of COX-2 by LPA and TNF-α in human primary cells.

## Conclusion

**Figure 5:**
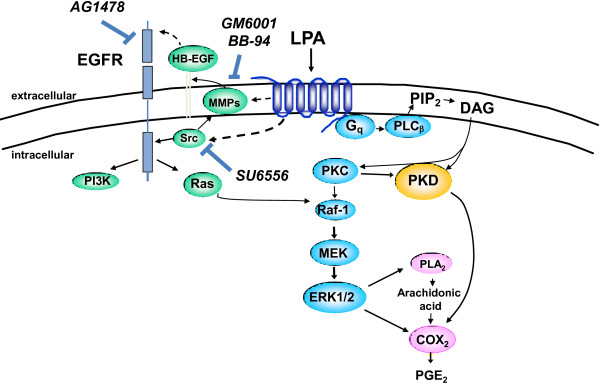
**LPA-mediated transactivation of upregulated EGFR enhances COX-2 expression in colonic myofibroblasts*.*** We have previously demonstrated that TNF-α potentiates LPA-mediated COX-2 expression via PKD [8], and that TNF-α induces upregulation of EGFR expression and signaling in human colonic myofibroblasts [11]. Here, we demonstrate cross talk between these two signaling pathways through LPA-mediated transactivation of upregulated EGFR, leading to enhanced COX-2 expression in colonic myofibroblasts. A summary of the signaling interactions is illustrated in graphical form.

Myofibroblasts, TNF-α, LPA, EGFR, and COX-2 have all been strongly and independently implicated in the development of colorectal cancer [10,17-19]. This study describes a novel interaction (Figure 5) that links myofibroblasts with these mediators and may partially explain how cross talk interactions initiated by TNF-α may incite both an inflammatory and a cytoprotective response that predisposes to colitis-associated cancer. The present study supports the notion that TNF-α utilizes EGFR signaling to mediate physiologic effects [11,20], and raises the possibility that targeted inhibition of one pathway may influence the biologic function of the other. Given the critical importance of TNF-α and EGF on intestinal inflammation, mucosal repair, and the development of cancer, the identification of signaling interactions between them may provide invaluable insight in the pathophysiology of colitis-associated cancer.

Furthermore, mounting evidence supports a causal relationship between LPA-mediated signaling and cancer progression [21], particularly in the context of colitis-associated cancer [22]. This connection was reinforced by the discovery that autotaxin, an enzyme known to promote tumor invasion and metastasis, acts by producing LPA in the tumor microenvironment [17]. LPA has been linked to a variety of cancers, including colorectal cancer [22-24], as well as to the production of COX-2 and prostaglandin secretion [22,25] by stromal cells of the gastrointestinal tract [26]. In a similar fashion, aberrant activation of EGFR signaling has been implicated in the development and progression of many human cancers [27-29]. The results presented here identify a novel mechanism of cross talk involving LPA and TNF-α which stimulates EGFR signaling and COX-2 expression, through LPA-induced transactivation in myofibroblasts. Future investigations will also evaluate potential cross talk mechanisms between TNF-α, LPA, and EGFR in the regulation of COX-1. Enhanced EGFR signaling within the intestinal stroma may play an important role in the development and progression of inflammation-associated cancer. The upregulation of EGFR signaling within the tumor stroma deserves closer attention and may be a novel target for anticancer therapies.

## Competing interests

The authors declare that they have no competing interests.

## Authors’ contributions

JY conceived of the study, participated in the study design, performed statistical analysis and drafted the manuscript. CP carried out cell culture work and Western blot experiments. WN carried out cell culture work, performed Western blot experiments, and isolated primary human myofibroblasts. JS was involved in study design. ER participated in study design and coordination, and with drafting of the manuscript. All authors read and approved the final manuscript.

## Pre-publication history

The pre-publication history for this paper can be accessed here:

http://www.biomedcentral.com/1471-230X/13/90/prepub
